# Discovery and Molecular Basis of a Diverse Set of Polycomb Repressive Complex 2 Inhibitors Recognition by EED

**DOI:** 10.1371/journal.pone.0169855

**Published:** 2017-01-10

**Authors:** Ling Li, Hailong Zhang, Man Zhang, Mengxi Zhao, Lijian Feng, Xiao Luo, Zhenting Gao, Ying Huang, Ophelia Ardayfio, Ji-Hu Zhang, Ying Lin, Hong Fan, Yuan Mi, Guobin Li, Lei Liu, Leying Feng, Fangjun Luo, Lin Teng, Wei Qi, Johannes Ottl, Andreas Lingel, Dirksen E. Bussiere, Zhengtian Yu, Peter Atadja, Chris Lu, En Li, Justin Gu, Kehao Zhao

**Affiliations:** 1 China Novartis Institutes for BioMedical Research, Shanghai, China; 2 Novartis Institutes for BioMedical Research, Cambridge, Massachusetts, United States of America; 3 Novartis Institutes for BioMedical Research, Basel, Switzerland; 4 Novartis Institutes for BioMedical Research, Emeryville, California, United States of America; Centre National de la Recherche Scientifique, FRANCE

## Abstract

Polycomb repressive complex 2 (PRC2), a histone H3 lysine 27 methyltransferase, plays a key role in gene regulation and is a known epigenetics drug target for cancer therapy. The WD40 domain-containing protein EED is the regulatory subunit of PRC2. It binds to the tri-methylated lysine 27 of the histone H3 (H3K27me3), and through which stimulates the activity of PRC2 allosterically. Recently, we disclosed a novel PRC2 inhibitor EED226 which binds to the K27me3-pocket on EED and showed strong antitumor activity in xenograft mice model. Here, we further report the identification and validation of four other EED binders along with EED162, the parental compound of EED226. The crystal structures for all these five compounds in complex with EED revealed a common deep pocket induced by the binding of this diverse set of compounds. This pocket was created after significant conformational rearrangement of the aromatic cage residues (Y365, Y148 and F97) in the H3K27me3 binding pocket of EED, the width of which was delineated by the side chains of these rearranged residues. In addition, all five compounds interact with the Arg367 at the bottom of the pocket. Each compound also displays unique features in its interaction with EED, suggesting the dynamics of the H3K27me3 pocket in accommodating the binding of different compounds. Our results provide structural insights for rational design of novel EED binder for the inhibition of PRC2 complex activity.

## Introduction

Polycomb repressive complex 2 (PRC2) is an essential component of the epigenetic machinery, regulating gene repression primarily through methylation of histone H3K27, which leads to changes in chromatin structure and thus exerts influence on key biological processes such as embryonic development. Abnormal activation of PRC2 due to genetic mutations in its catalytic subunit EZH2 has been widely studied in lymphoma malignancy [1–7], which serves as the basis for targeting PRC2 as novel therapeutic approach in treating malignant cancers such as follicular lymphoma (FL) and diffuse large B-cell lymphoma (DLBCL). We and others have discovered potent PRC2 inhibitors derived from a common pyridone scaffold which compete with the cofactor SAM for EZH2 binding [8–12]. These inhibitors showed high selectivity to PRC2 *in vitro*, reduced global H3K27 methylation in cells, engaged in modulating EZH2 dependent gene expression, and more importantly, inhibited tumor growth in xenograft animal models. The current leading molecule, EPZ6438, is now in phase 2 clinical trials for DLBCL and FL [10].

The minimal core PRC2 complex is composed of three protein components: the catalytic subunit EZH2 (Enhancer of zeste homolog 2), the WD40 domain comprising EED (embryonic ectoderm development), and the VEFS domain comprising SUZ12 (suppressor of zeste 12). EED and SUZ12 are essential for EZH2 activity, as depletion of either SUZ12 or EED completely abrogates PRC2 function, leading to H3K27me3-devoid chromatin and subsequent gene activation [13–15]. Indeed, the negative-stained electron microscopy structure of PRC2 suggested EED, the c-terminal part of SUZ12, and EZH2 form the catalytic core complex of globular architecture, which could be distanced from RBAP48 and the N-terminal SUZ12 interaction [16]. Recent crystal structures of the EZH2-EED-SUZ12 ternary complex further revealed the molecular basis of the PRC2 core complex assembly. In these structures, the EED subunit is wrapped around by EZH2, and SUZ12 is sandwiched between EED and the SET domain of EZH2 to form a catalytically active complex [17–19]. Importantly, the allosteric activation of PRC2 by H3K27me3 and JARID2-K116me3 peptides is attributed to the stabilization of the stimulation recognition motif (SRM), leading to the conformational change in the SET-I of the EZH2 SET domain [19]. The recognition of H3K27me3 by EED and the resulting increase of the methylase activity are believed to be crucial for the cellular function of PRC2 in propagating the repressive marks at the chromatin loci [20]. Therefore, blocking this process would be an attractive approach in modulating PRC2 functions.

EED contains seven WD40 repeats and adopts a canonic β-propeller architecture with the ‘top’ portion for H3K27me3 binding and its ‘bottom’ portion anchored to the N-terminal helix of EZH2 [20–22]. A stabilized alpha-helix mimetic of the EZH2 peptide has been shown to selectively inhibit H3K27 trimethylation of PRC2 *in vitro* and suppresses the growth of MLL-AF9 leukemia cells via disrupting the EZH2/EED interaction [23]. However, the low potency and the weak cellular permeability of such a peptide inhibitor will likely limit its therapeutic applications.

We recently disclosed a novel allosteric PRC2 inhibitor EED226 which binds to the H3K27me3 pocket of EED and completely regressed tumor growth in the xenograft mouse model [24]. Here, we further report the identification of several additional small molecular inhibitors of PRC2 (including the parental compound of EED226) that bind to the H3K27me3 binding-pocket of EED. These compounds were discovered from a high-throughput screen using the PRC2 enzymatic assay as the primary screening assay. Subsequent characterization demonstrated that the mechanism of inhibition (MOI) of these compounds to PRC2 complex is indeed through EED binding. These compounds inhibit not only the H3K27me3-stimulated PRC2 activity, but also the basal activity *in vitro*. The co-crystal structures of the compounds bound to EED demonstrate a common and yet dynamic “induced fit” in the H3K27me3 pocket of EED with significant conformational change of the aromatic cage residues. This set of novel EED binders provide an attractive starting point for developing novel PRC2 allosteric inhibitors.

## Materials and Methods

### Protein Expression and Purification

To prepare protein for crystallization, EED, comprising residues 76–441 was cloned into pGEX-KG with an N-terminal GST tag and was expressed in E.coli strain BL21-CodonPlus(DE3)-RIL. The cells were sonicated and centrifuged to collect the supernatant. The soluble EED protein in the supernatant was purified by affinity chromatography using of Glutathione Sepharose 4B resin (GE) and subsequently cleaved by tobacco etch virus (TEV) protease at 4°C overnight, except for the EED protein used for the EED396 co-structure, which was instead cleaved by thrombin protease. The cleaved protein was then flowed through a Ni^2+^-NTA column (QIAGEN) to remove the His-tag and the protease, and was followed by a gel filtration column (Superdex 75, 10/60 from GE). The purified EED was then concentrated to 8 mg/ml and stored in 50 mM Tris pH 8.0, 100 mM NaCl, 5 mM DTT and 10% glycerol (v:v).

### High Throughput Screening and Hit Triage

The EZH2 HTS biochemical assay was developed into a homogeneous time resolved fluorescence (HTRF) format. H3K27me0 peptide (comprising residues 21–44) and biotinylated at its C-terminus was used as substrate. EZH2 activity was detected by measuring di-methylated peptide formation via the TR-FRET energy transfer between an Europium cryptate (the donor) labelled rabbit monoclonal anti-dimethyl H3K27 antibody and XL665 (the acceptor) which was conjugated to streptavidin. The final assay used 2.5 μM SAM, 0.5 μM biotinylated unmethylated Histone H3 peptide (residues 21–44) and 50 nM EZH2 in the Design of Experiment (DOE) optimized reaction buffer containing 20 mM Tris pH 8, 1 mM DTT, 0.1% Triton X-100, 0.1% BSA, 1 mM EDTA. The assay was then miniaturized into a 1536-well plate format, with a final reaction volume of 4 μL and a final detection volume of 8 μL. After incubation at ambient temperature for 4 hrs, a stop-and-detection mix comprise of 200 μM SAH, 1 nM Eu-labelled anti-dimethyl histone H3K27 antibody (Cisbio), and 80 nM streptavidin conjugated XL665 in 100 mM Hepes, pH 7, 0.2% BSA and 0.8 M potassium fluoride was added to stop the reaction and detect product formation with a ViewLux plate reader, using excitation at 320 nm and emission at 590 nm and 665 nm.

To perform the full-deck screen, 1536-well assay plates (Nunc black 1536-well plate, Cat. No. 264711) were dotted with 20 nL of compound at a concentration of 2 mM in each well in columns 1–44 of the plate. 2 μL of substrate mixture containing 2.5 μM, S-adenosylmethionine (SAM), 0.5 μM biotinylated unmethylated Histone H3 peptide was then dispensed into the plate (column 1–46; columns 47 and 48 were dispensed with a substrate mix comprised of 200 μM, S-adenosylhomocysteine (SAH) at pH 7. Following this, 2 μL of EZH2 complex was dispensed into the plate to start the reaction. After 4 hrs of incubation, 4 uL of 2x of the HTRF detection mix (80 nM SA-XL665 and 1 nM Eu-anti dimethyl Histone H3K27 antibody—final conc.) was added to all wells. The plate was then read using the Viewlux plate reader after 1.5 hrs incubation.

The confirmation assay followed the same protocol as in the primary screen as described above. For the counterscreen, compounds were mixed with 0.2 μM biotinylated dimethyl histone H3K27 (21–44) peptide, 0.3 μM biotinylated unmethylated Histone H3 (21–44) peptide and 2.5 μM SAM. The plates were allowed to incubate for 1 hr, then the stop-and-detection solution described above was added to each well and the plates were read in the Viewlux following 1.5 hrs incubation.

Approximately 1.4 million compounds were screened in the EZH2 HTRF assay in 1536-well plates. The screening assay signal-to-background ratio was approximately 6-fold and the median Z’ was 0.75. 11,765 compounds at 30% or higher inhibition were identified as primary hits. After chemoinformatic triage of these initial hits and confirmation of the inhibition, 2,911 compounds were confirmed. From the confirmation and counterscreen data, 1,967 compounds were selected and tested in dose response titration from 15 μM to 0.1 μM in a 1:2 serial dilution series. Of these, 1,405 compounds produced valid dose response curves. These hits were further validated in the LC-MS orthogonal assay, mechanism-of-action assay, cellular H3 trimethylation assay, biophysical assay and HMT profiling. Data and chemoinformatic clustering analyses were performed to classify the compounds as SAM competitive, EED binding and other mechanism of actions and to prioritize these chemical scaffolds.

### IC50 Determination by LC-MS Based PRC2 Biochemical Assay

This assay employs liquid chromatography mass spectrometry technology using peptide or nucleosome as substrate and then detects the formation of the co-product SAH [11, 25]. To assess the compounds potency in the H3K27me0 peptide (21–44) based PRC2 enzymatic assay, compounds were serially diluted 3-fold in neat DMSO to obtain a total of twelve screening concentrations. Compounds at each concentration were then transferred by Mosquito into a 384-well Perkin Elmer ProxiPlate 384 plus plates. The typical reaction was 12 μL in volume, included 1 μM SAM (at Km), 1.5 μM H3K27me0 peptide (at Km) and 10 nM PRC2 in the assay buffer composed of 20 mM Tris-HCl, pH 8.0, 0.01% Triton X-100, 0.5 mM DTT and 0.1% BSA. The reaction was stopped by adding 3 μL quench solution containing 2.5% TFA and 320 nM SAH-d4 (Cayman chemical).

The enzyme activity of PRC2 were measured based on the production of SAH using LC-MS. SAH-d4 was used as an internal standard (IS) control for quantification. Briefly, samples were run on an API 4000 LC/MS/MS system. Liquid chromatography was performed on a Chromolith Fast Gradient HPLC column (RP-18e, 25-2mm, Merck). The column was connected to the mass spectrometer through a 6-port valve. The turbo ion electrospray was operated in the positive-ion mode. The m/z values for the parent ions of SAH and SAH-d4 are 385.1 and 389.1, respectively; the m/z values for the daughter ions of SAH and SAH-d4 are both 136.1. Mobile phase A was 0.02% FA (v:v) and 2% methanol (v:v) in water while mobile phase B is 0.1% FA (v:v) in acetonitrile. The injection volume was 4 μL and the auto-sampler was kept at 4°C. The eluents between 0.4 and 1.0 minute were diverted to the mass spectrometer for analysis. The ratio of the SAH peak area/IS peak area vs SAH concentration was plotted to generate the normalization factor of SAH. The production of SAH from the enzymatic reaction was derived from the standard curve of SAH. The lower limit of our system for the detection of SAH is around 1–2 nM, while the linear range can reach up to 400 nM.

The protocol for the PRC2 LC-MS assay with recombinant nucleosome core particles (NCP) as substrate was very similar to the H3K27me0 peptide based LCMS assay described above except that NCP was used as substrate. Meanwhile, the activation peptide H3K27me3 (21–44) was included to stimulate the PRC2 activity for the nucleosome substrate.

### EED-H3K27me3 (19–33) AlphaScreen Competition Binding Assay

To assess the compounds potency in the EED-H3K27me3 AlphaScreen competition binding assay, compounds were serially diluted 3-fold in DMSO to obtain a total of twelve concentrations. Compounds at each concentration were then transferred into 384-well Perkin Elmer ProxiPlate 384 plus plates. 8 μL of solution containing 30 nM His-EED (residues 1–441) protein and 15 nM biotin-H3K27me3 (residues 19–33) peptide in buffer containing 25 mM HEPES, pH 8, 0.02% Tween-20, and 0.5% BSA, were added to the wells and then incubated with compound for 20 min. An AlphaScreen detection beads mix (Perkin Elmer) was prepared immediately before use by mixing nickel chelate acceptor beads and streptavidin donor beads in a 1:1 ratio into the buffer described above. 4 μL of detection beads mix were then added to the plate and incubated in the dark at the room temperature for 1 h. The final concentration of both the donor and acceptor beads was 10μg/mL. Plates were read using an EnVision system (PerkinElmer) using the AlphaScreen setting adapted for optimal signal detection with a 615 nm filter following sample excitation at 680 nm. The emission signal at 615 nm was used to quantify a compound’s inhibition. AlphaScreen signals were normalized based on the reading coming from the positive control (DMSO control) and negative control (no EED protein added control) to give percentage of activity remaining. The data were then fit to a dose response equation using the program Helios (Novartis) to determine the IC50 values.

Each compound was counterscreened to determine if it interfered with the AlphaScreen beads. Compounds were diluted as described in the preceding section, and the assay was performed by adding 12 μL of 10 nM Biotin-miniPEG-His6 peptide in the above buffer and incubated for 20 min at room temperature prior to addition of the acceptor and donor beads. The plates were then incubated for 1 h at room temperature in the dark before being read on the EnVision system (Perkin Elmer).

### ITC Binding Analysis

ITC experiments were performed on an Auto ITC200 (Microcal) at 25°C. The ITC sample cell was filled with 10 μM PRC2 in titration buffer containing 25 mM HEPES pH8.0, 150 mM NaCl, and 1% DMSO. 40 μL of 100 μM compounds in the same titration buffer was loaded in the ITC syringe. 19 injections, each of 2 μL, was injected into the ITC cell at 150 s intervals. Binding constant (K) and binding enthalpy (ΔH) determinations were obtained from the fit to the experimental data using a one-site model.

### Surface Plasmon Resonance (SPR) Binding Analysis

SPR experiments were performed on a Biacore T100 instrument (GE Health Science) and data analysis was conducted with the BiaEvaluation software (GE Health Science). SA sensor chips were obtained from GE Health Science. Biotinylated recombinant EED (residues 40–441) in buffer containing 25 mM HEPES (pH 7.5), 50 mM NaCl and 0.01% TWEEN-20 (HBS) was immobilized to an SA chip to reach an immobilization level of approximately 5000–6000 RU. Each compound in DMSO stock solution was serial diluted with DMSO before a final dilution with HBS to yield a 2% DMSO solution in HBS with concentrations ranging from 1.56 to 50 μM. For each binding cycle, the compound sample was injected with a flow rate of 40 μL/min and a 60s sample injection followed with a dissociation time of 90 s. Due to the completed dissociation of compounds, no regeneration was performed. Given the fast-on/fast-off binding features of compounds tested, steady-state mode was performed to determine the binding affinities (K_D_) by plotting the compound-binding response (RU) against the compound concentration.

### Crystallization and Data Collection

To generate the crystal structure of EED-EED396 complex, an apo-EED (thrombin-cleaved) crystal was grown using sitting drop vapor diffusion with a reservoir containing 3.3 M Na formate and 0.01 M Yttrium chloride hexahydrate. The apo-crystal was soaked with 1mM EED396 for 7 hours. The TEV-cleaved EED protein was mixed with EZH2 peptide (residues 40–68, EBD) with molar ratio of 1: 2.5. For compounds EED709, EED666 and EED210, the EED-EBD peptide complex was crystallized in 0.1 M Tris pH 8.0 and 16% PEG 8000 (w:v) using the sitting drop vapor diffusion method at 20°C. 10 mM β-Nicotinamide mononucleotide (NMN) was added to the drop as an additive to improve the crystal quality. Crystals grew overnight and were then soaked in a drop containing 1 mM compound containing reservoir for 2 days before flash froze frozen in liquid nitrogen. The EBD peptide NH2-KSMFSSNRQKILERTEILNQEWKQRRIQPV-CO2H, corresponding to human EZH2 (residues 40–68) was chemically synthesized (GL Biochem). For the EED-EBD-EED162 complex structure, 1 mM of compound was added to the mixture of EED-EBD and incubated on ice for approximately 1 hour. Co-crystallization was set up using the sitting drop diffusion method at 20°C with 0.1 M Bis-Tris pH 6.0, 0.2 M MgCl2, and 20% PEG 3350 (w:v) as a precipitant. 30% glycerol (v:v) was used as a cryoprotectant. Diffraction data was collected at the Shanghai Synchrotron Radiation Facility (SSRF) beamline BL17U1 and processed using HKL2000 [26] or XDS [27].

### Structure Determination and Refinement

The protein structure of the mouse EED in complex with mouse EZH2 peptide (Protein Data Bank code 2QXV), which is identical to human EED, was used to create a search model for molecular replacement using Molrep in the CCP4 suite [28]. The compounds were manually built in COOT [29] and the structures was further refined using REFMAC5 [30] and BUSTER [31]. The final structures were checked using the programs PROCHECK [32]. The statistics of the structure refinement and the quality of the final model are summarized in Table 1.

**Table 1:** Data collection and statistics for the structure of EED -inhibitor complexes.

Crystal	EED-EED396	EED-EBD-EED666	EED-EBD-EED709	EED-EBD-EED162	EED-EBD-EED210
PDB code	5H13	5H14	5H15	5H19	5H17
***Data collection***					
Space Group	*P* 2_1_ 2_1_ 2	*P* 2_1_ 2_1_ 2	*P* 2_1_ 2_1_ 2	*C* 2 2 2_1_	*C* 2 2 2_1_
Unit Cell Parameters [Å]	150.7, 46.0, 51.8	93.8, 178.4, 50.5	93.5, 179.1, 50.6	50.5, 91.0, 179.2	50.6, 92.3, 180.5
α,β,γ [°]	90.00 90.00 90.00	90.00 90.00 90.00	90.00 90.00 90.00	90.00 90.00 90.00	90.00 90.00 90.00
Contents of ASU^1^					
Protein Molecules	1 EED	2 EED, 2 EBD	2 EED, 2 EBD	1 EED, 1 EBD	1 EED, 1 EBD
Ligand Molecules	1 EED396	2 EED666	2 EED709	1 EED162	1 EED210
Resolution [Å]	30.00–1.90(2.00–1.90)^2^	50.00–1.90(1.97–1.90)^2^	50.00–2.30(2.38–2.30)^2^	179.17–1.90 (1.91–1.90)^2^	90.24–2.27(2.28–2.27)^2^
Unique Reflection	229130 (4298)	67346 (6659)	38289 (3812)	33040 (18317)	21839 (6817)
Completeness [%]	99.7 (100.0)	99.5 (100)	96.4 (98.1)	100.0 (100.0)	99.8 (99.5)
Redundancy	5.1 (5.8)	6.7 (6.8)	5.6 (5.6)	7.3 (7.37)	7.1 (7.2)
R_merge_ [%]	7.7 (36.2)	8.3 (53.1)	12.1 (45.4)	13.4 (43.6)	10.3 (66.9)
I/σ(I)	4.3 (2.1)	23 (5.1)	13.1 (3.6)	11.8 (5.2)	14.4 (3.6)
**Refinement**					
Resolution [Å]	28.36–1.90 (1.97–1.90)	40.27–1.90 (1.95–1.90)	43.16–2.27 (2.33–2.27)	25.19–1.90 (1.96–1.90)	30.00–2.30 (2.42–2.30)
No. of Reflections	29081(2807)	67285(4760)	38238(2486)	32764(2746)	19203(2746)
**Completeness [%]**	99.6(99.6)	99.2(99.2)	95.1(95.1)	99.2(99.2)	99.9(99.9)
**R**_**work**_[%]	19.8 (21.8)	19.3 (23.1)	18.5 (20.1)	17.9 (22.0)	19.2 (21.8)
**R**_**free**_^3^[%]	22.9 (24.8)	22.3 (25.6)	23.6 (28.8)	21.5 (30.4)	23.9 (28.5)
Wilson B [Å^2^]	29.75	23.67	30.26	24.55	46.63
**Average B-factor [**Å^2^**]**					
Overall	36.9/	33.8	26.8	27.5	42.4
Protein/Ligands/Water	36.6/43.8/41.4	30.2/42.4/37.4	26.6/27.5/30.4	27.0/22.7/35.8	42.3/40.7/43.9
No. of solvent molecules	96	412	319	228	121
V_M_^4^(%solvent)	2.03(39.51)	2.30(46.61)	2.31(46.70)	2.24(45.18)	2.29(46.4)
CC(Fo-Fc)(Fo-Fc free)	0.94/0.92	0.93/0.92	0.93/0.90	0.94/0.92	0.92/0.89
*rmsd* bonds[Å]/angles[°]	0.010/1.11	0.010/1.06	0.010/1.13	0.010/1.05	0.010/1.15
Ramachandran (core)	96.6%	96.6%	96.1%	97.4%	95.5%
Clash score	3.26	2.05	3.64	2.08	1.93
MolProbity score	1.86	1.68	1.97	1.51	1.78

^1^Asymmetric Unit;

^2^numbers in parenthesis are for highest resolution shell;

^3^test set uses 5% data;

^4^Matthews Coefficient

### Druggability Score Calculation

The protein chain A of each structure is extracted. All steps shown in Maestro Protein Preparation Wizard were followed to prepare the EED protein and ligands. Ligand protonation states were set at pH 7.0+/-2.0 using Epik, and protein protonation state was set at pH 7.0 using PROPKA. H-bond assignment was optimized by protassign, including exhaustive sampling and minimizing hydrogens of altered species at neutral pH. Finally, all ligand and water molecules were removed, and all crystal structures were superposed to EED-H3K27me3 (PDB ID: 3IIW) structure.

Schrodinger SiteMap was used to calculate the Dscore of the ligand binding site of each complex structure. The “Evaluate a single binding site region” task of SiteMap is selected, the region around H3K27me3 peptide from EED-H3K27me3 (PDB ID: 3IIW) structure plus 6Å buffer was chosen to be examined as the binding site.

## Results

### Development of the PRC2 Enzymatic Assays and the Identification of H3K27me3-Competive Inhibitors

PRC2 enzymatic assays using the recombinant five-member PRC2 complex and either the H3(21–44) peptide or the recombinant mono-nucleosome core particle (NCP) as the substrate were developed. These PRC2-catalyzed reactions were studied in detail by quantitatively measuring the formation of SAH with LC/MS/MS [11, 25]. In addition, when the H3 peptide was used as the substrate, the formation of mono-, di- and tri-methylated H3 peptides were also monitored semi-quantitatively.

**Fig 1:**
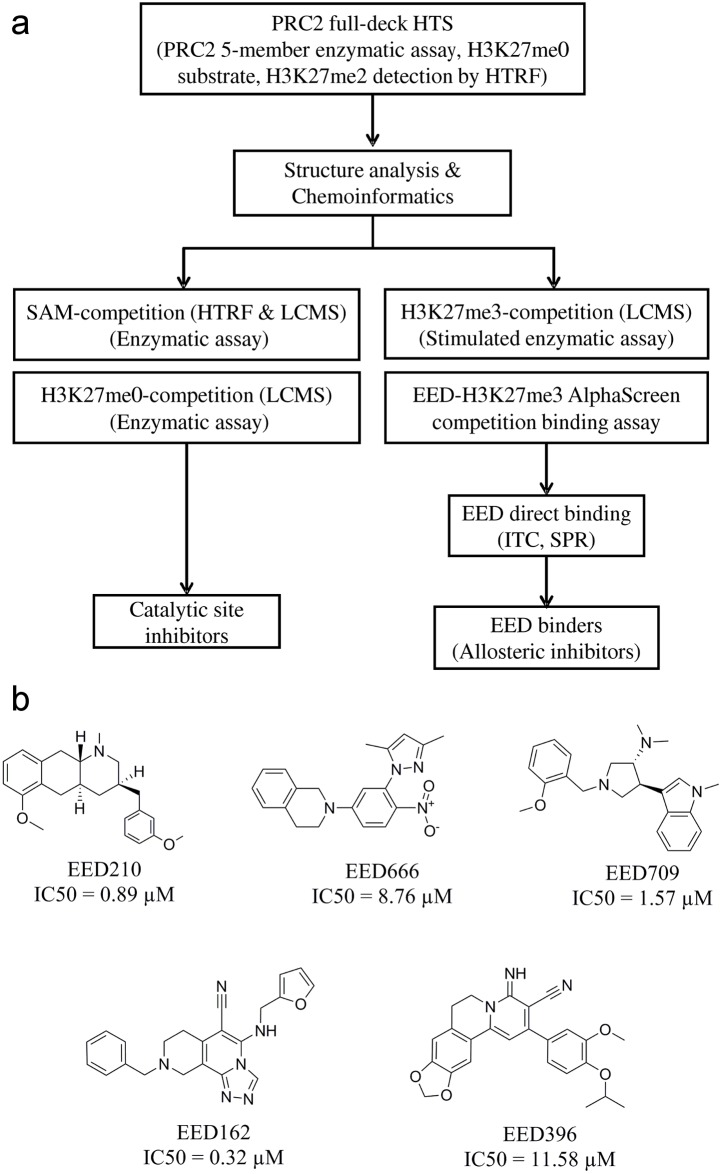
Identification of allosteric PRC2 inhibitors through EED binding. **a.** Flowchart of EEDi identification and validation from PRC2 high throughput screening. **b.** Chemical structures of five identified SAM non-competitive inhibitors. The IC50 values were determined using an EED-H3K27me3 AlphaScreen competition binding assay.

The basal activity of PRC2 and the effect of the stimulatory H3K27me3 peptide were studied. When the H3K27me0 peptide was used as the substrate, PRC2 had robust activity which could only be slightly increased by the stimulatory peptide. However, when recombinant NCP was used as the substrate, the PRC2 activity was much lower. This activity could be markedly increased by adding the H3K27me3 stimulatory peptide in a dose dependent manner with half-maximum stimulation (K_act_) at 1.0 μM [24]. The peptide-based assay (in the absence of the H3K27me3 stimulatory peptide) was then converted into a Homogeneous Time Resolved Fluorescence (HTRF) assay using a Europium-labelled rabbit monoclonal antibody against dimethylated H3K27. Balanced conditions were chosen: S-adenosylmethionine (SAM) concentration was set at K_m_ and peptide concentration was set below its K_m_. After further optimization and miniaturization, this HTRF assay was used to screen a collection of about 1.4 million compounds in 1,536-well plates (Fig 1a).

More than a thousand hits identified from the screen were subsequently confirmed. Compounds with IC50 < 50 μM were then selected for further validation. To understand their MOI and to identify the real PRC2 inhibitors among them, SAM-competition experiments were first performed in the same HTRF assay using two different SAM concentrations (at 1x SAM-K_m_ and 10x SAM-K_m_ concentrations). Several compounds were identified as SAM-competitive inhibitors, among them was the parental compound of pyridone scaffold EI1 [11]. However, the majority of these HTS hits behaved as SAM non-competitive inhibitors.

**Fig 2:**
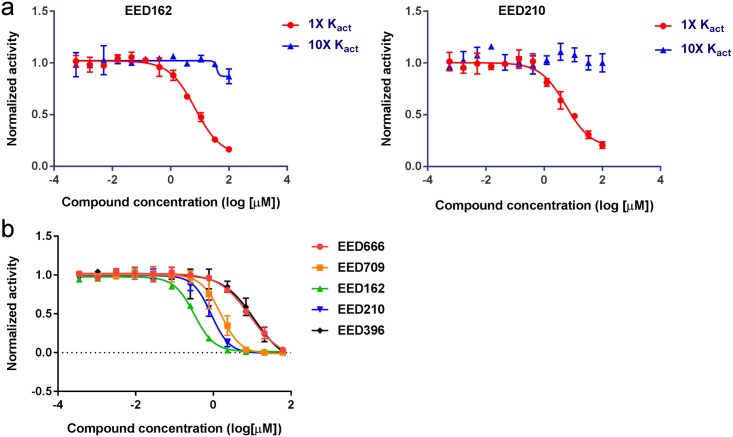
EED inhibitors compete with H3K27me3 peptide in both enzymatic and binding assays. **a**. EED inhibitors compete with H3K27me3 in NCP based PRC2 enzymatic assays. The assay was carried out at 1 x and 10 x K_act_ for the stimulatory H3K27me3 peptide and the concentration of SAM and nucleosome were kept at K_m_. Inhibitors demonstrated significantly reduced potency at a higher concentration of H3K27me3 peptide. **b**. EED inhibitors compete with H3K27me3 in EED-H3K27me3 AlphaScreen binding assay (competition mode). All compounds reduced the AlphaScreen signal in a dose dependent-manner.

To further validate the SAM non-competitive inhibitors, several additional assays were then developed (see flowchart Fig 1a). We first focused on the H3K27me3 pocket on EED. We reasoned that although no H3K27me3 peptide was included in the primary H3K27me0 peptide-based HTS assay, there is still a possibility that low molecular weight compounds could bind to this pocket and negatively regulate the PRC2 activity. To identify such compounds, H3K27me3-competition experiments using the recombinant NCP-based enzymatic assay were performed. Compounds were tested in the presence of 1x or 10x K_act_ of H3K27me3 peptide, and their IC50s under these two conditions were compared. Compounds that bind to the H3K27me3 pocket should show an increased IC50 in the presence of a higher concentration of H3K27me3 peptide. In addition to the enzymatic assay, we also developed a binding assay to measure the interaction between His-tagged recombinant EED protein and a biotinylated H3K27me3 peptide using the Amplified Luminescence Proximity Homogenous Assay Screen (AlphaScreen) [33, 34]. Binding of Biotin-H3K27me3 peptide to His-EED protein brings the streptavidin-coated donor beads and anti-His antibody coated acceptor beads into close proximity and thus a luminescent signal is produced. Disruption of EED-H3K27me3 interaction by H3K27me3 pocket binders will result in the signal reduction. Using these two assays, we identified multiple H3K27me3 competitive compounds from the SAM non-competitive hits. Five representative scaffolds are shown in Fig 1b. These compounds showed a dramatically increased IC50 in the enzymatic assays when the H3K27me3 concentration was increased from 1x K_act_ to 10x K_act_ (Fig 2a and S1c Fig). Furthermore, in the AlphaScreen binding assay, they reduced the H3K27me3 binding signal to the basal level (with IC50 ranges from 0.3 μM to 11.6 μM, Figs 1b and 2b), suggesting that they can fully antagonize the binding of the H3K27me3 peptide to EED.

### Characterization of the H3K27me3-Competitive Inhibitors

**Fig 3:**
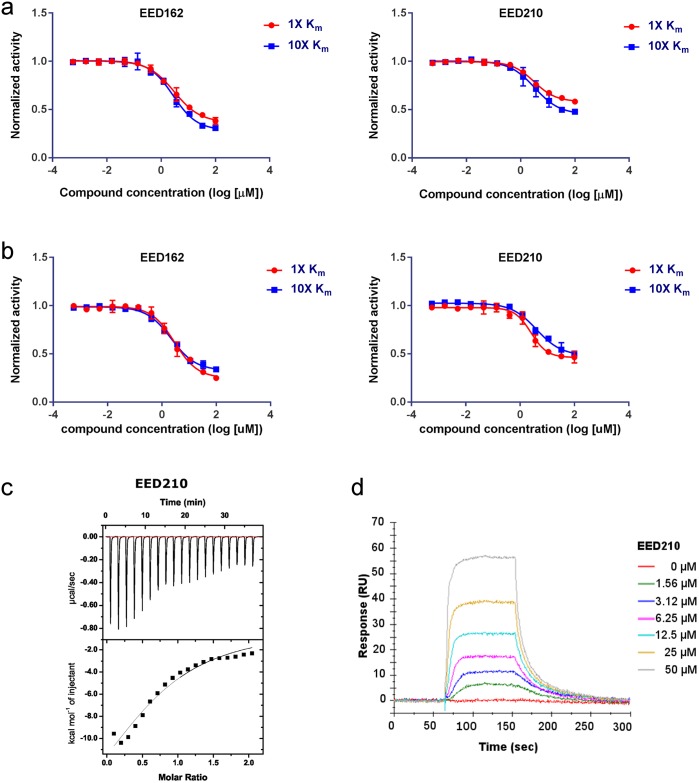
EED inhibitors are non-competitive with SAM or H3K27me0 peptide. **a.** EED inhibitors are non-competitive with SAM. Enzymatic assays were carried out at 1 x and 10 x SAM with H3K27me0 in excess. There is no IC50 shift when increasing SAM concentration. **b**. EED inhibitors are non-competitive with H3K27me0. Enzymatic assays were carried out at 1 x and 10 x H3K27me0 with SAM in excess. There is no IC50 shift when increasing H3K27me0 concentration. **c**. Binding affinity determination of EED210 to EED by ITC. The stoichiometry of binding between EED210 and EED was approximately 1:1 molar ratio with N = 0.78. The enthalpy change is -20.14 ±5.29 Kcal/mol and the entropy change is -46.5cal/mol/deg. **d**. Concentration dependent SPR analysis of EED210 binding to EED (residues 40–441).

By using H3K27me0 (21–44) peptide as a substrate and measuring the basal activity of PRC2, we further studied the effect of SAM and H3K27me0 substrate peptide concentration on compound potency. As shown in Fig 3a and 3b and S1a and S1b Fig, none of them showed a change in potency when either SAM or the substrate H3K27me0 peptide was increased to 10x K_m_. All these compounds behave as SAM non-competitive and peptide substrate non-competitive.

We then carried out biophysical studies to validate whether these compounds indeed directly bind to EED. Both isothermal calorimetry (ITC) and surface plasmon resonance (SPR) assay were established for this purpose. Fig 3c shows the ITC data for the binding of EED210 to recombinant EED with a calculated K_D_ of 28 μM. Similarly, EED210 also showed a good binding signal to immobilized EED (40–441) in the SPR assay with a binding affinity of 35.1μM (Fig 3d). We could not perform high quality ITC and SPR analysis for the rest of compounds because of their limited solubility. Despite the limited solubility, all five compounds were put into crystal trials for final confirmation because of their strong enzymatic and AlphaScreen binding data (see the next section).

We then tested the selectivity of these compounds in a histone methyltransferases (HMT) panel in *vitro*. All compounds are highly selective inhibitors of PRC2 and showed no inhibitory activity towards other HMTs (IC50 >100 μM) in a panel of 22 such enzymes (S1 Table). It is worth noting that all five compounds showed similar inhibitory activities to both EZH2 and EZH1 harboring PRC2 complexes. This is not surprising as EED is the common component in both complexes.

Taken together, these data clearly showed that we have identified a novel class of PRC2 inhibitors. This class of compounds allosterically inhibit the basal activity of PRC2 by directly binding to the EED subunit (thereafter these compounds are termed as EEDi). In addition, they compete with H3K27me3 peptide for the binding to EED and thus inhibit the H3K27me3-stimulated PRC2 catalytic activities. Their MOI is thus distinct from previously reported SAM-competitive PRC2 complex inhibitors.

### Molecular Basis of EEDi Interaction with EED

To elucidate the binding modes of EED396, EED666, EED709, EED162 and EED210, we determined the crystal structure of EED in complex with each respective compound at high resolution (Table 1). The crystal structures were solved using a EED-EZH2 peptide (40–68) complex, except for EED396, which was determined using apo-EED. Each compound in the structure is well defined in both unbiased *F*o-*F*c and refined 2*F*o-*F*c electron density maps (S2 Fig) The EZH2 peptide-bound EED structure is highly similar to the apo-EED structure with root mean-square deviation (RMSD) of 0.58 Å of 317 pairs of Cα atoms between the two structures. Therefore, all five complex structures can be directly compared to each other.

**Fig 4:**
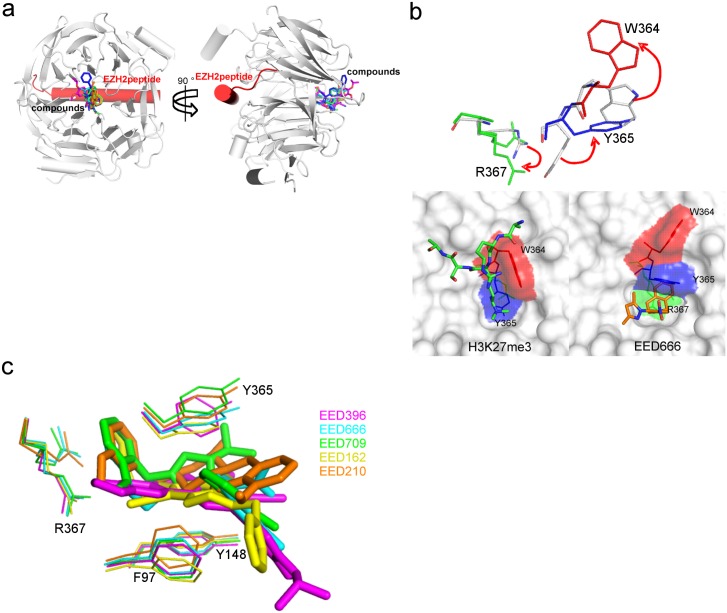
The crystal structures of H3K27me3 competitive inhibitors binding to EED. **a.** Structures of EED-EZH2 peptide in complex with EED396, EED666, EED709, EED162 and EED210. The five structures are aligned and shown in the same view. The EZH2 peptide is highlighted as red cylinder. **b.** A representative highlight of the conformational change of Arg367, Trp364, and Tyr365, in comparison of the EED666 bound (Arg 367 in green, Tyr365 in blue, and Trp364 in red) in and H3K27me3 bound EED structures (top); below, comparison of EED666-bound EED pocket (right) with H3K27me3-bound pocket (left); EED is shown as a surface and colored white. H3K27me3 peptide is shown as ball-and-stick in green color; for clarity, only the surface of residues Arg367 (green), Tyr365 (blue) and Trp364(red) are highlighted. **c**. The dynamic conformational changes of Arg367, Tyr365, Tyr148 and Phe97 in inhibitor bound EED structures.

The overall structures of the inhibitor bound EED complexes are very similar with RMSD of Cα atoms ranging from 0.17Å to 0.36Å (Fig 4a). As illustrated by the biochemical results, all five compounds do indeed bind directly in the H3K27me3 pocket (Fig 4a). The overall structures of these complexes also share high similarity with the H3K27me3 peptide bound EED (PDB: 3IIW) and EED in the PRC2 complex (PDB: 5HYN) (S3a Fig). However, they exhibit significant induced conformational changes for residues Trp364 and Tyr365 (with a movement of the respective side-chain of more than 5Å) (Fig 4b), moderate changes for residues Arg367, Glu238, and Met256, and minor changes to residues Phe97 and Tyr148 (S3c Fig). Together, these changes result in the formation of a much deeper pocket as compared to the original aromatic cage found when H3K27me3 peptide is bound (Fig 4b).

The key residues that govern the shape of the pocket are Tyr365, Tyr148, Phe97 and Arg367. The three aromatic residues of the aromatic cage for H3K27me3 recognition [20, 21] form the wall of the newly induced pocket. Their side chains delineate the width of the pocket and adopt slightly different conformations to accommodate different compounds (Fig 4c), suggesting the dynamics of the pocket in compound binding. Arg367 is at the bottom of the cavity. It delineates the depth of the pocket and functions as the “gate keeper” restricting access of the compounds into the deep β-propeller center of EED. It is interesting to note that the side-chain of Arg367 is flexible and can adopt at least two distinct rotamers (S2f Fig). Overall, the cavities induced by the binding of these five compounds are much larger and deeper than the aromatic cage present in H3K27me3-bound EED (S2 Table). Furthermore, the cavity is dynamic and its size can change to accommodate the binding of different compounds (S2 Table). This is largely due to the flexibility of the side chains of the above-mentioned residues. As expected, this compound-induced deep pocket has a higher druggability score (Dscore) [35] of around 1 on average compared to that of 0.67 when the peptide is bound.

**Fig 5:**
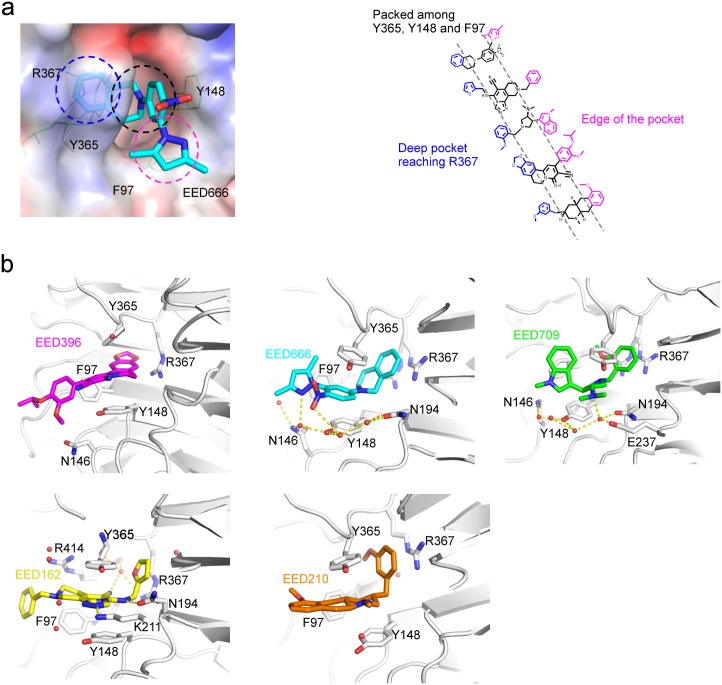
The molecular basis of H3K27me3 competitive inhibitors recognition by EED. **a.** Electrostatic surface potentials of EED666 binding pocket (left); Blue—positive charge, red—negative charge; The blue, black and pink dashed circles indicate the deep pocket, aromatic packing region and the edge of pocket, respectively; Five compounds are aligned to underline the common binding features (right). Chemical groups embedded in the deep pocket are colored blue; chemical groups packed against Tyr365, Tyr148 and Phe97 are colored black; chemical groups located in the edge of the pockets are colored pink. Chemical groups involved in three binding regions are divided by two dashed lines. **b.** Binding mode of each co-structure. Interacting residues in EED are labeled and shown as sticks. Water molecules are shown as red sphere. Yellow dashed lines are hydrogen bonds.

The interactions of all five compounds with EED share common features. First, they each harbor a chemical moiety that can reach deep into the bottom of the pocket and interact with Arg367 (Fig 5a and 5b). The side-chain of Arg367 adopts a conformation that allows it directly contact the various moieties. The phenyl moiety of EED666, the furan moiety of EED162, and the methoxyphenyl moieties of EED709 and EED210 all make cation-π stacking interactions with the guanidinium group of the Arg367 side chain, with the characteristic face-to-face orientation. As an exception, the benzodioxole moiety of EED396 engages in an edge-to-face interaction with the Arg367 side-chain. Second, the central rings of each of the five compounds are positioned between Phe97, Tyr365 and Tyr148, inducing conformational changes in all of these three aromatic residues to accommodate the compounds. While the central rings of EED666, EED162 and EED396 form π-π stacking interactions with the side-chains of Phe97, Tyr365 and Tyr148, those of EED709 and EED210 are involved in cation- π and Van der Waals contacts. Lastly, all five compounds contain chemical moieties extending to the edge of the pocket. These moieties still maintain interactions with EED, even though they are located in the edge (Fig 5a and 5b).

Despite sharing the aforementioned common features, each compound also displays unique features in its interaction with EED, further suggesting the dynamics of the H3K27me3 binding pocket in responding to the binding of diverse compound (Fig 5b). For instance, the dioxolane of EED396 makes a unique contact with Arg367 in the deep pocket. The nitro group of the nitrophenyl moiety and the pyrazole nitrogen of EED666 both interact with Asn146, Tyr148 and Asn194 through a water-mediated hydrogen bonding network (Fig 5b). In addition, the tetrahydroisoquinoline moiety of EED666 induces the rearrangement of Tyr365 to make the nitrophenyl moiety interact with Tyr365 and Tyr148 through a cation/ π - π interaction. The pyrrolidine-amine moiety of EED709 forms interactions with Asn146, Tyr148, Asn194 and Glu237 via a water-mediated hydrogen bonding network (Fig 5b). The cyano group in the core of EED162 is hydrogen-bonded to the side-chain of Arg367 and the main-chains of Arg414 and Tyr365 via a water-mediated network. The triazolo-pyridine core and amide linker of EED162 form hydrogen bonds with the side chains of Lys211 and Asn194, respectively. The amine group of the octahydrobenzo-quinoline moiety of EED210 forms cation-π stacking with Tyr148 and Tyr365. Overall, these residues including Arg367, Tyr148, Tyr365 and Phe97 displayed various conformations in response to interactions with the each of the five compounds. Therefore, optimizing interactions with these residues can potentially rigidify these conformations and make enthalpically favorable interactions to improve the binding affinities of these compounds.

## Discussion

In this study, we describe the identification of a set of novel small molecule inhibitors of PRC2. We have demonstrated that these inhibitors bind to EED and compete with H3K27me3 binding. The crystal structures revealed that these compounds induce dramatic conformational changes of the residues in the aromatic cage of EED. As expected, the H3K27me3-stimulated PRC2 activity can be completely inhibited by this class of compounds. In the published PRC2 complex structure, the H3K27me3 peptide is sandwiched between the SRM of EZH2 and EED. Thus, the H3K27me3 peptide appears to stabilize the rather flexible SRM [17–19]. The change in the SRM results in the conformational change in other parts of EZH2 (mainly in SET-I) leading to its full activation [17–19, 36]. The five EED binders apparently cannot stimulate PRC2 activity (they actually inhibit the PRC2 basal activity). Therefore, they are likely unable to stabilize the interaction of SRM of EZH2 with EED, even though they do bind in the same position in EED as the H3K27me3 peptide. However, it remains possible that LMW compounds may exist that could mimic H3K27me3 peptide to stimulate PRC2 activities.

The five compounds we report here inhibit the basal activity of PRC2, in particular when the peptide H3K27me0 (21–44) is used as substrate. However, the detailed molecular mechanism of this allosteric inhibition remains to be determined. We did not find significant structural differences (outside the aromatic cage region) between the EED in the hPRC2 complex (PDB: 5HYN) and the EED in the EED-EEDi complexes (S3b Fig). As the allosteric regulation of PRC2 activity seems to be intricate, it is possible that subtle changes in EED upon EEDi binding would be sufficient to affect the activity of the complex.

Proteins that recognize methylated lysines in histone tails play important roles in regulating many cellular functions through modulating chromatin-mediated signaling and epigenetic regulation of gene expression [37, 38]. Dysregulated activities of these reader proteins have been reported in many diseases including cancer. The majority of the methylated lysine readers are considered to be less druggable compared to the acetylated lysine readers, the bromodomain proteins [35]. For example, EED was believed to have a low druggability potential, with a druggability score of below 0.7 [35]. Our discovery of small HTS hits (all with MW less than 500 daltons) capable of binding to EED at micromolar potency provides a convincing example that EED indeed is druggable. The dynamics of the H3K27me3 pocket in EED as demonstrated in our structures may be the main reason why EED seems to be readily druggable by LMW inhibitors.

The identification of multiple hits against the same binding pocket on EED enabled us to define the common feature of this LMW compound binding pocket as well as some unique interactions for each individual compound. Collectively, this information has been very helpful for guiding the lead optimization effort. The manuscripts for the optimization results for EED210 and EED162 will be published separately [24, 39, 40]. In conclusion, we have discovered a new class of PRC2 allosteric inhibitors through binding to EED. Our work provides new chemical starting points that are suitable for the development of therapeutics targeting PRC2.

## Supporting Information

S1 Fig Characterization of mode of action of EEDi. (a) EED inhibitors in H3K27me3 peptide competition experiment. (b) EED inhibitors in SAM competition experiment. (**c**) EED inhibitors in H3K27me0 competition experiment.
(TIF)

S2 Fig Electron density maps of EEDis. The grid in blue color showing the refined 2*F*o-*F*c (σ = 1) map of the inhibitors. The grid in limon color showing the unbiased *F*o-*F*c (σ = 3) map of the inhibitors. a, EED396. b. EED666. c. EED709, d. EED162. e. EED210. f. Refined 2*F*o-*F*c map (σ = 1) showing the two rotamer conformations of Arg367 in the EED-EED709 complex structure.
(TIF)

S3 Fig Comparison of EEDi bound EED structures with other EED structures. a. Superimposition of EED666-EED complex structure (EED in grey, EZH2 peptide in red and EED666 in cyan with sphere mode) with that of EED-H3K27me3 complex (EED in orange; PDB code: 3IIW). b. Superimposition of EED666-EED complex structure with that of EED in the PRC2 complex (EED in limon; PDB code: 5HYN). c. Per residue RMSD (Å) between EED-EEDi complexes and EED-H3K27me3 complex (PDB code: 3IIW).
(TIF)

S1 Table Selectivity of inhibitors against a panel of histone methyltransferases. (DOCX)

S2 Table The compound bound H3K27me3 pocket size and the druggability score. (DOCX)
