# Supplementary figures and images for: Discovery and Molecular Basis of a Diverse Set of Polycomb Repressive Complex 2 Inhibitors Recognition by EED
Source: PLoS One. 2017 Jan 10;12(1):e0169855. doi: 10.1371/journal.pone.0169855 (PMC5224880; doi:10.1371/journal.pone.0169855)

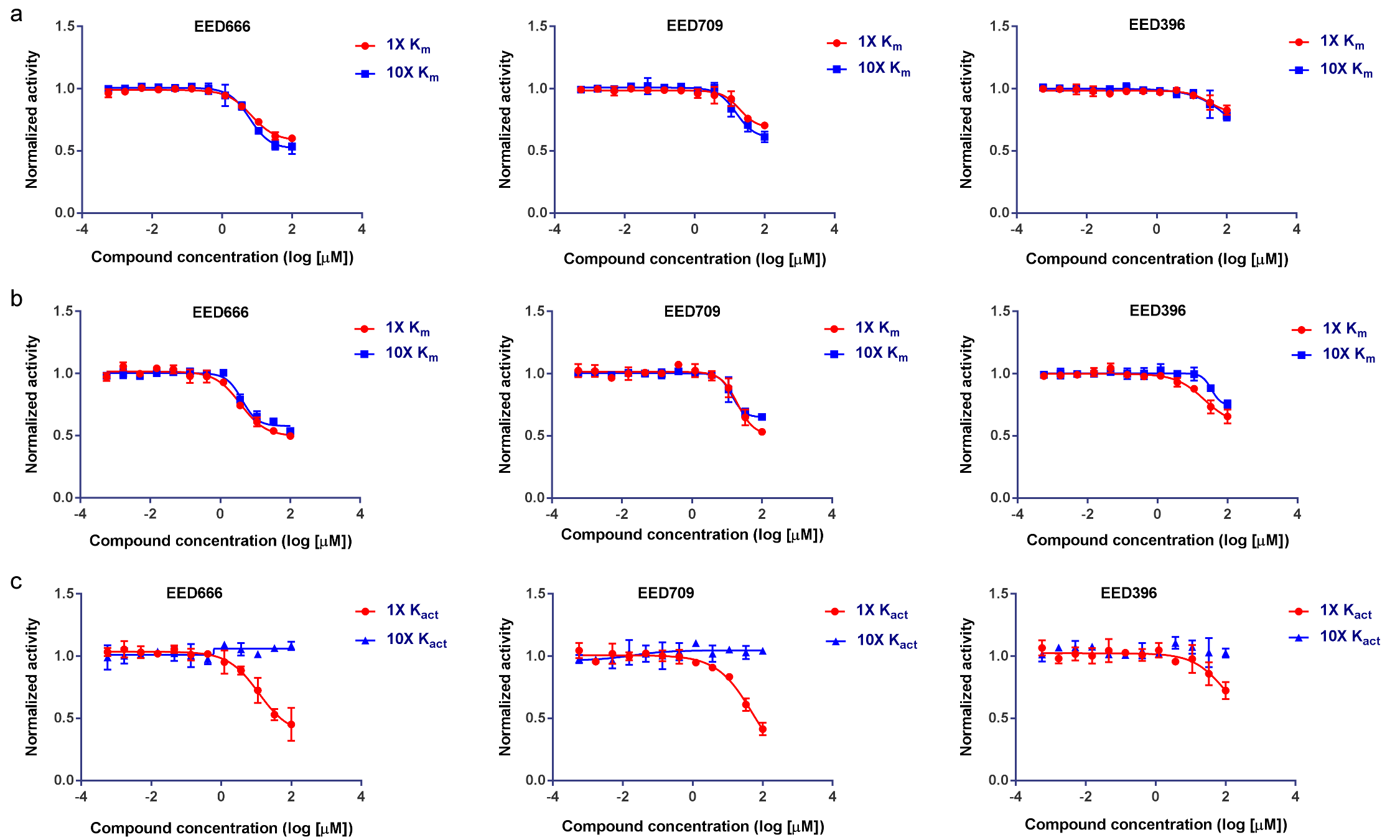

Supplement: S1 Fig — (a) EED inhibitors in H3K27me3 peptide competition experiment. (b) EED inhibitors in SAM competition experiment. (c) EED inhibitors in H3K27me0 competition experiment. (TIF) [file pone.0169855.s001.tif]

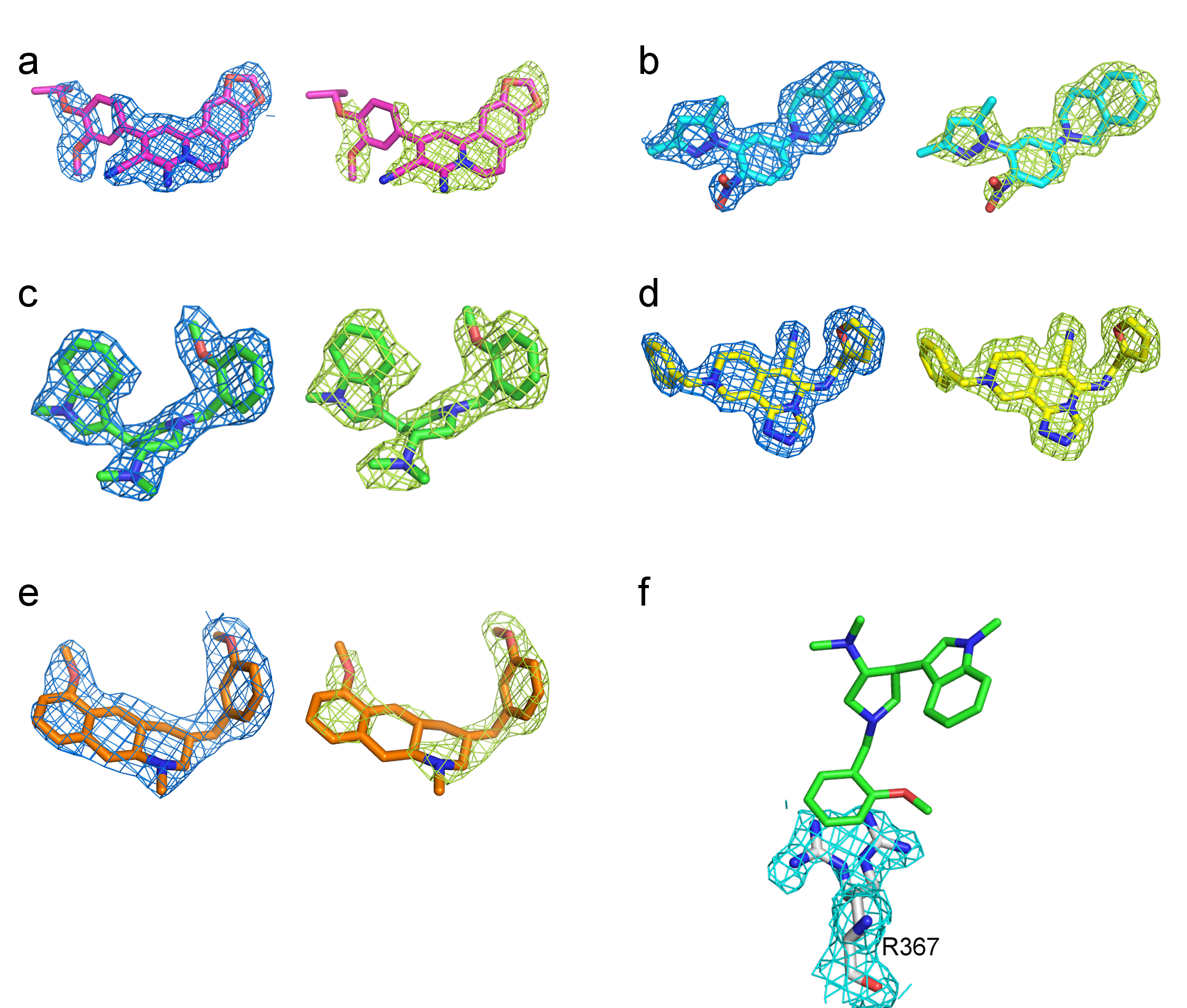

Supplement: S2 Fig — The grid in blue color showing the refined 2Fo-Fc (σ = 1) map of the inhibitors. The grid in limon color showing the unbiased Fo-Fc (σ = 3) map of the inhibitors. a, EED396. b. EED666. c. EED709, d. EED162. e. EED210. f. Refined 2Fo-Fc map (σ = 1) showing the two rotamer conformations of Arg367 in the EED-EED709 complex structure. (TIF) [file pone.0169855.s002.tif]

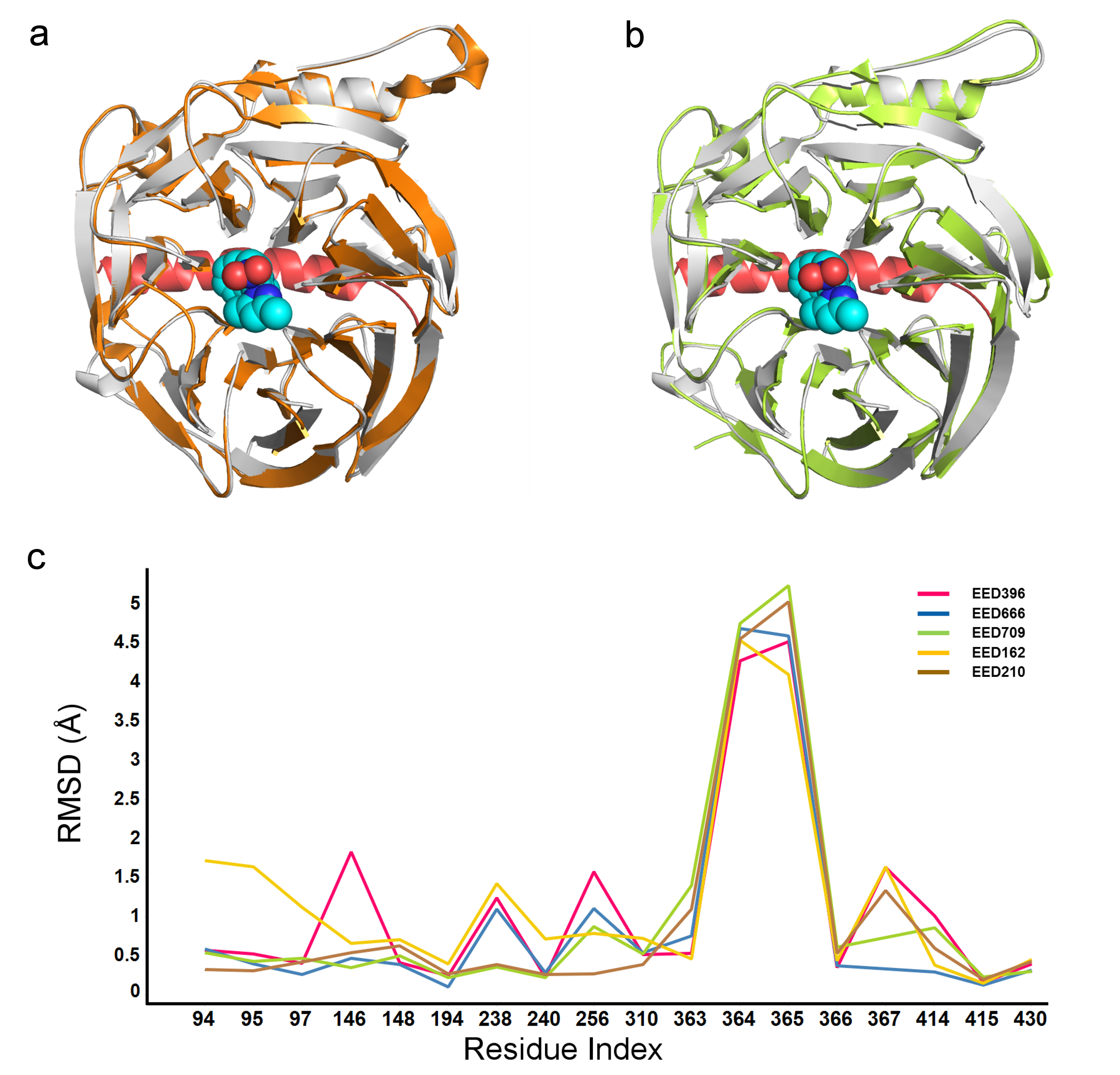

Supplement: S3 Fig — a. Superimposition of EED666-EED complex structure (EED in grey, EZH2 peptide in red and EED666 in cyan with sphere mode) with that of EED-H3K27me3 complex (EED in orange; PDB code: 3IIW). b. Superimposition of EED666-EED complex structure with that of EED in the PRC2 complex (EED in limon; PDB code: 5HYN). c. Per residue RMSD (Å) between EED-EEDi complexes and EED-H3K27me3 complex (PDB code: 3IIW). (TIF) [file pone.0169855.s003.tif]
