# Supplementary material for: Discovery and Molecular Basis of a Diverse Set of Polycomb Repressive Complex 2 Inhibitors Recognition by EED
Source: PLoS One. 2017 Jan 10;12(1):e0169855. doi: 10.1371/journal.pone.0169855 (PMC5224880; doi:10.1371/journal.pone.0169855)
Supplement: S1 Table — (DOCX) [file pone.0169855.s004.docx]

| **Enzyme** | **EED210^a^** | **EED162^a^** | **EED709^b^** | **EED396^b^** | **EED666^b^** |
| --- | --- | --- | --- | --- | --- |
|  | **IC50 (µM)** | **IC50 (µM)** | **IC50 (µM)** | **IC50 (µM)** | **IC50 (µM)** |
| EZH2 | 2.8 | 2.2 | 98 | 87 | 99 |
| EZH1 | 2.8 | 5.1 | 62 | 70 | 66 |
| MLL | >100 | >100 | >100 | >100 | >100 |
| SETD6 | >100 | >100 | >100 | >100 | >100 |
| SMYD3 | >100 | >100 | >100 | >100 | >100 |
| SMYD2 | >100 | >100 | >100 | >100 | >100 |
| Set7/9 | >100 | >100 | >100 | >100 | >100 |
| SetD8 | >100 | >100 | >100 | >100 | >100 |
| Suv39H2 | >100 | >100 | >100 | >100 | >100 |
| G9a | >100 | >100 | >100 | >100 | >100 |
| ESET | >100 | >100 | >100 | >100 | >100 |
| NSD2 | >100 | >100 | >100 | >100 | >100 |
| NSD1 | >100 | >100 | >100 | >100 | >100 |
| NSD3 | >100 | >100 | >100 | >100 | >100 |
| SETD2 | >100 | >100 | >100 | >100 | >100 |
| Dot1L | >100 | >100 | >100 | >100 | >100 |
| PRMT1 | >100 | >100 | >100 | >100 | >100 |
| PRMT3 | >100 | >100 | >100 | >100 | >100 |
| CARM1 | >100 | >100 | >100 | >100 | >100 |
| PRMT5/MEP50 | >100 | >100 | >100 | >100 | >100 |
| PRMT8 | >100 | >100 | >100 | >100 | >100 |
| DNMT1 | >100 | >100 | >100 | >100 | >100 |
|  |  |  |  |  |  |

**S1 Table. Selectivity of inhibitors against a panel of histone methyltransferases.**

Note: for EZH2, EZH2/EED/SUZ12/RBAP48/AEBP2 was used in the assay; for EZH1, EZH1/EED/SUZ12/RBAP48/AEBP2 was used in the assay; for MLL, MLL/WDR5/RBBP5/ASH2L/ was use in the assay. a, H3K27me0 peptide was used as substrate for both EZH2 and EZH1 activity; b, H3K27me1 peptide was used as substrate for both EZH2 and EZH1 activity.

- All HMT reactions were performed as described previously.
- ref: [Proc Natl Acad Sci U S A.](http://www.ncbi.nlm.nih.gov/pubmed/23236167) 2012 Dec 26;109(52):21360-5.
