# Supplementary material for: Discovery and Molecular Basis of a Diverse Set of Polycomb Repressive Complex 2 Inhibitors Recognition by EED
Source: PLoS One. 2017 Jan 10;12(1):e0169855. doi: 10.1371/journal.pone.0169855 (PMC5224880; doi:10.1371/journal.pone.0169855)
Supplement: S2 Table — (DOCX) [file pone.0169855.s005.docx]

**S2 Table**. The compound bound H3K27me3 pocket size and the druggability score.

| Structures | Volume (Å^3^) | Depth (Å) | Dscore |
| --- | --- | --- | --- |
| EED-H3K27me3  (PDB: 3IIW) | 88.5 | 5 (me3.NZ->Y365.CZ) | 0.679 |
| EED-EED396 | 156.0 | 8.3 (me3.NZ->R367.CZ) | 1.067 |
| EED-EED666 | 159.4 | 8.0 (me3.NZ->R367.CZ) | 1.106 |
| EED-EED709 | 175.9 | 7.8 (me3.NZ->R367.CZ) | 1.075 |
| EED-EED162 | 119.7 | 8 (me3.NZ->R367.CZ) | 0.914 |
| EED-EED210 | 160.8 | 8.8 (me3.NZ->R367.CZ) | 1.100 |

- All HMT reactions were performed as described previously.
- ref: [Proc Natl Acad Sci U S A.](http://www.ncbi.nlm.nih.gov/pubmed/23236167) 2012 Dec 26;109(52):21360-5.
